# Long Noncoding RNAs, New Critical Regulators in Cancer Immunity

**DOI:** 10.3389/fonc.2020.550987

**Published:** 2020-10-30

**Authors:** Minjie Wu, Peifen Fu, Lei Qu, Jian Liu, Aifu Lin

**Affiliations:** ^1^ Breast Center of the First Affiliated Hospital, School of Medicine, Zhejiang University, Hangzhou, China; ^2^ MOE Laboratory of Biosystem Homeostasis and Protection, College of Life Sciences, Zhejiang University, Hangzhou, China; ^3^ Reproductive and Developmental Biology Laboratory, National Institute of Environmental Health Sciences, Durham, NC, United States

**Keywords:** cancer, cancer immunity, lncRNA, immunotherapy, combined therapy

## Abstract

Long noncoding RNAs (lncRNAs) play crucial roles in various aspects of cellular functions. Recent studies have revealed that lncRNAs are critical players in the immune system by modulating immune cell differentiation and functions, particularly in cancer immunity. Here we systematically summarize how lncRNAs are involved in different processes of the cancer immunity cycle, including immune cell differentiation, proliferation, trafficking, and infiltration. Moreover, the limitations of the current understanding of lncRNA’s functions in cancer immunity are described, such as the complexity of the cancer immunity system, the inclusive functions of lncRNAs in this system, and the associated immune response. In sum, the comprehensive investigation of the roles of lncRNAs in cancer immunity aids in cancer diagnosis and therapies.

## Introduction

With the advance of the human genome sequence, more than 90% of the human genome transcribed RNAs lack protein-coding functions. Nearly 75% among them are noncoding RNAs (ncRNAs), which are divided into two groups: small ncRNAs (<200nt) and long ncRNAs (lncRNAs) (≥200nt) (1). Although lncRNAs cannot directly encode proteins due to the lack of open reading frames, they can control gene transcription and expression by regulating transcriptional activators or repressors, different components of transcription reaction (e.g., RNA polymerase II), and DNA duplex (2). LncRNAs also regulate post-transcription, such as mRNA splicing (3, 4) and translation (5). What is more, they regulate epigenetic modification, including DNA methylation (6, 7), histone acetylation (8), and ubiquitination (9). LncRNAs powerfully function in various cellular activities. For example, lncRNAs play indispensable roles in the progression of various cancers in past years, such as breast cancer (10, 11), prostate cancer (12), gastric cancer (13), and lung cancer (14–16). Of note, lncRNAs have been found to be the key players in regulating cancer immunity. In this review, we aim to summarize the findings of lncRNAs functions in cancer immunity as well as their clinical impacts.

## Cancer Immunity Cycle

**Figure 1:**
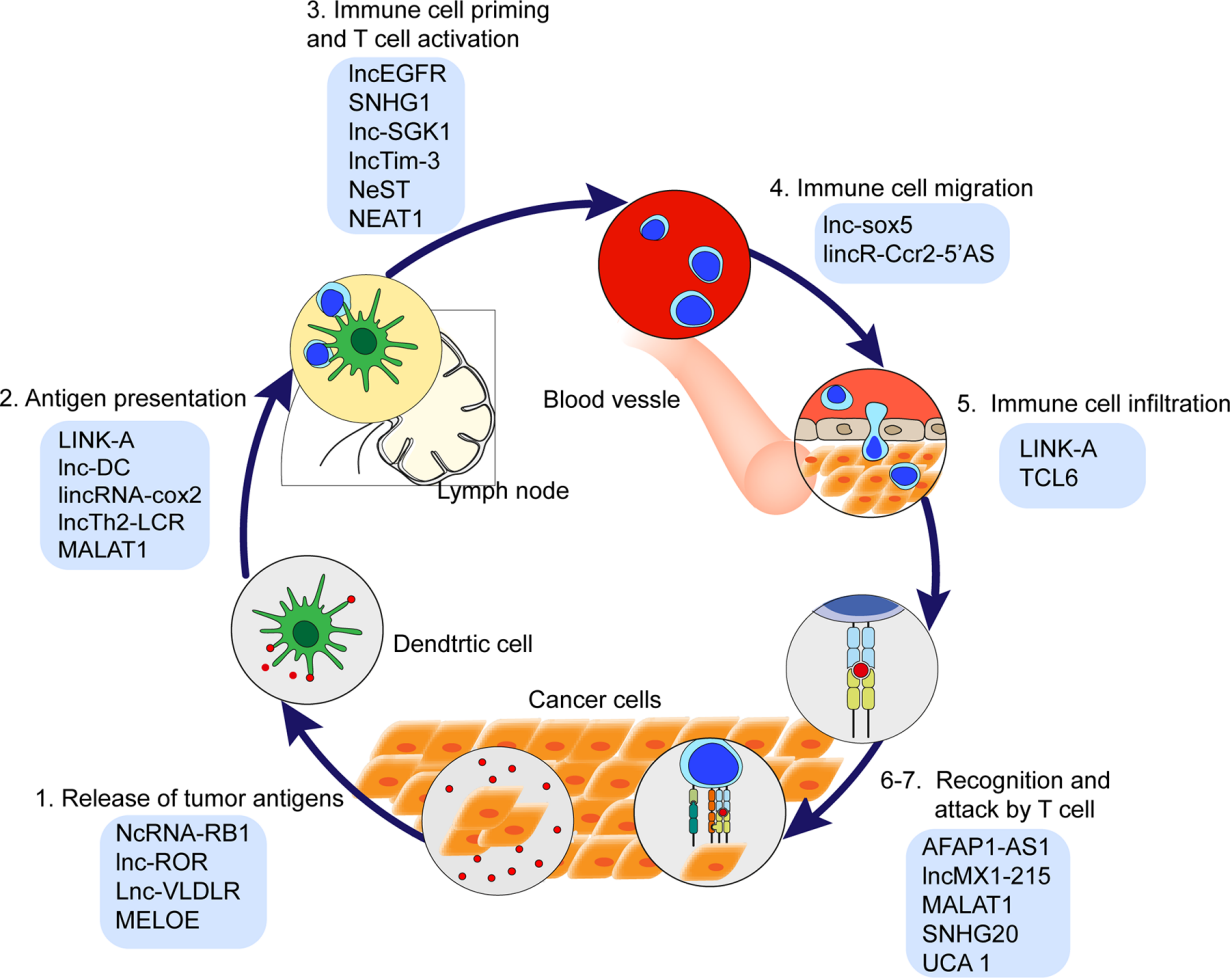
The cancer immunity cycle and the lncRNAs involved in each step of cancer immunity cycle.

**Figure 2:**
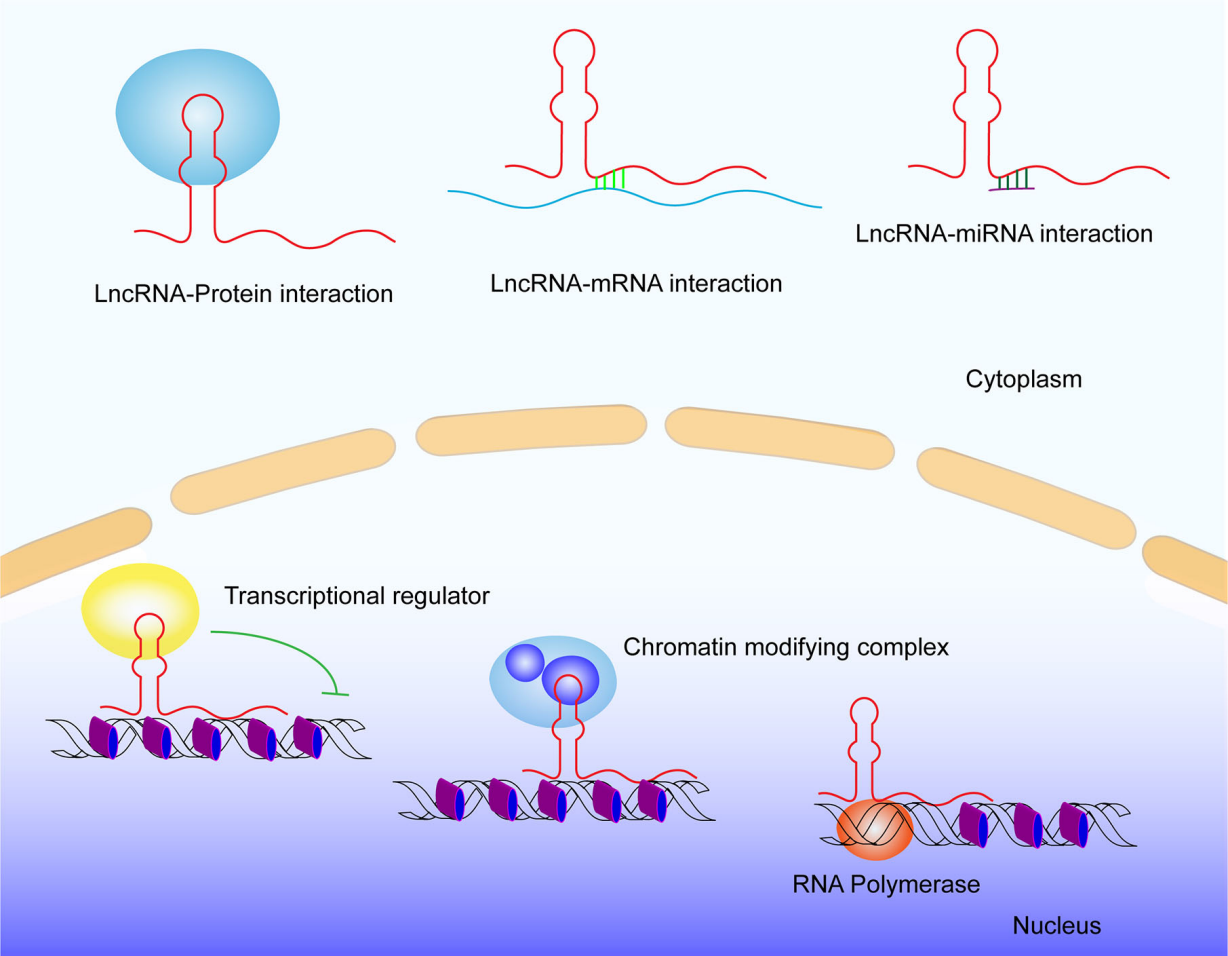
General lncRNA mechanisms. LncRNA interacts with DNA, RNA, and Proteins.

The cancer immunity cycle is defined as stepwise events effectively controlling cancer cell growth by immune system. This cycle is arbitrarily divided into seven steps (**Figure 1**): cancer cells release antigens (Step 1); antigens are captured by dendritic cells (DCs) (Step 2); DCs with captured antigens migrate to lymph node and prime with T cells to activate tumor-specific cytolytic CD8^+^ T cells (Step 3); the cytolytic T cells migrate from lymph node into blood vessels (Step 4); immune cells infiltrate into tumor stroma (Step 5); the cytolytic T cells recognize tumor cells (Step 6); T cells kill cancer cells (Step 7) (17, 18). Recently, lncRNAs have been revealed as key regulators in the cancer immunity cycle (**Figure 2** and **Table 1**), including the impact on the epigenetic and transcriptional profiles of immune cells.

**Table 1:** LncRNAs involved in cancer immunity and their functions.

Steps of cancer immunity cycle	LncRNAs	Functions of lncRNAs
**1. Release of antigens**	NcRNA-RB1	NcRNA-RB1 inhibits the expression of CALR, prevent tumor cells release “killing me” signal (19).
	Lnc-ROR	Lnc-ROR promotes the proliferation of CD133+ cells through TGF-β in HCC (20).
	Lnc-VLDLR	Lnc-VLDLR expressed in EV promotes tumor cell death and releases noe-antigen (21).
	MELOE	MELOE increase immunogenicity to be recognized by TCLs (22).
**2. Antigen presentation**	LINK-A	LINK-A inhibits antigen presentation in breast cancer (23).
	lnc-DC	Lnc-DC promotes differentiation of monocytes to dendritic cells (24, 25).
	LncTh2-LCR	LncTh2-LCR regulates the expression of Th2 cytokines, IL-4, IL-5, and IL-13 (26).
	LincRNA-cox2	LincRNA-cox2 inhibits chemokines (Ccl5, Cx3c11) and cytokines (Ccrl) receptors expression (27).
	MALAT1	MALAT1 inhibits the production of TNF-α and IL-6 to repress antigen presentation (28).
**3. Immune cell priming and T cell activation**	lncEGFR	LncEGFR links immunosuppression to cancer by promoting differentiation of Treg cells (9).
	SNHG1	SNHG1 impedes the immune escape by inhibiting the differentiation of Treg cells through promoting miR-448 expression and reduces the protein level of IDO (29).
	lnc-SGK1	Lnc-SGK1 promotes Th2 and Th17 differentiation by SGK1/JunB signaling.
	NEAT1	In HCC, NEAT1 promotes CD8^+^ T-cell apoptosis and enhances cytolysis (30).
	NeST	NeST promotes IFNG transcription to improve INF-γ production during CD8^+^ T cells activation (31).
	lncTim-3	LncTim-3 exacerbates CD8 T cell exhaustion (32).
**4. Immune cell migration**	lnc-sox5	Lnc-sox5 induces the down regulation of IDO1 to regulate infiltration and cytotoxicity of CD3^+^CD8^+^ T cells (33).
	lincR-Ccr2-5’AS	Knockdown of lincR-Ccr2-5’AS induces a dramatic migration of Th2 cells into lung in mice [34].
**5. Immune cell infiltration**	LINK-A	LINK-A, inhibits CD8^+^ T cell infiltration (23).
	TCL6	TCL6 promotes infiltration of CD8+ T cells, CD4+ T cells, neutrophils, DCs (35).
**6-7. Recognition and attack by T cell**	AFAP1-AS1	AFAP1-AS1 increase PD1 expression (36).
	LncMX1-215	LncMX1-215 promotes PD-L1 expression and immune escape (37).
	MALAT1	MALAT1 promotes PD-L1 expression and immune escape (38).
	SNHG20	SNHG20 promotes PD-L1 expression and immune escape (39).
	UCA 1	UCA 1 promotes PD-L1 expression and immune escape (40).

## lncRNAs in Tumor Antigen Release

Tumor antigen is an antigenic substance produced by tumor cells, especially dying tumor cells. It triggers the immune system to attack it (41, 42). Calreticulin (CALR), which is a calcium-binding chaperone and influences antigen presentation to cytotoxic T cells (43), is expressed in many cancer cells and promotes macrophages to engulf hazardous cancerous cells (44). In lung cancer, NcRNA-RB1, a lncRNA expressed from the RB1 promoter, inhibits the expression of CALR. It shows a tumor-inhibitor mechanism through impairing the “kill me” signal (19). Meanwhile, the efficiency of chemotherapy is associated with the expression of some lncRNAs besides the death of immune cells or released neoantigens. For example, Lnc-ROR, a stress-responsive lncRNA highly expressed in hepatocellular carcinoma (HCC) cells, promotes the proliferation of CD133^+^ cells by activating TGF-β pathway to weaken the effectiveness of chemotherapy drugs in HCC (20). Lnc-VLDLR (very low-density lipoprotein-receptor-VLDLR) is expressed in extracellular vesicles (EV) and promotes tumor cell death during chemotherapy, leading to increased chemotherapy efficiency (21). Taken together, the roles of lncRNAs in tumor antigen release are starting to be recognized.

## lncRNAs in Antigen Presentation

Antigen presentation is a vital immune process to trigger effective immune response. Activation of T cells depends on effective antigen presentation, including immunogenic signals and specific antigen presentation cells (APCs). LINK-A inhibits antigen presentation by specifically inhibiting antigen-presenting cell (APC) and decreasing CD8^+^ T cell abundance in basal-like breast cancer (45). DCs are antigen-presenting cells that act as the messengers between the innate and adaptive immune system. The lncRNA that is expressed exclusively in human DC (lnc-DC) has been identified as an lncRNA involved in the differentiation of monocytes to DCs. Lnc-DC mainly exists in the cytoplasm and activates DC transcription factor STAT3by binding with STAT3 and promoting STAT3 tyrosine-705 phosphorylation. During the differentiation of DCs, the transcript of lnc-DC is upregulated (24). Knockdown of lnc-DC impairs the expression of DC-specific genes regulating antigen presentation, T cell activation, and cell migration, such as *CD40*, *CD80*, *CD86*, and *CCR7* (24, 25). Moreover, deletion of lnc-DC in DC cells leads to impaired priming CD4^+^ T cells or secreting inflammatory cytokines after pathogen stimulation (24).

Moreover, lncRNAs regulate pro-inflammatory cytokines that are crucial to antigen presentation, such as IFN-γ and TNF-α (46–48). LincRNA-cox2 is an lncRNA located adjacent to Cox2. The study reported that silencing lincRNA-cox2 leads to increased expression of chemokines (Ccl5, Cx3c11) and cytokine receptors (Ccrl) (27). Moreover, lncRNA Th2 locus control region (lncTh2-LCR) regulates expression of Th2 cytokines, IL-4, IL-5, and IL-13 (26), and MALAT1 (Metastasis Associated Lung Adenocarcinoma Transcript 1) inhibits the production of TNF-α and IL-6 by inhibiting NF-kB DNA binding to gene promoters (28). More interestingly, lncRNAs also improve antigen presentation after being translated into short polypeptides. In melanoma, lncRNA MELOE, which is translated into MELOE-1, MELOE-2, and MELOE-3 by different translational approaches, shows the highest immunogenicity and can be recognized by tumor-infiltrating lymphocytes. MELOE is currently considered as a targeted specific antigen to improve the efficacy of melanoma immunotherapy (22). These findings establish the key roles of lncRNAs in antigen presentation.

## lncRNAs in Immune Cell Priming and Activation

Effective immune priming and activation are determined by the ratio of T effector cells and T regulatory (Treg) cells (49). Some lncRNAs have been demonstrated to regulate lymphocyte differentiation and activation (50–54). In HCC, lnc-epidermal growth factor receptor (lncEGFR) promotes Treg cells differentiation and suppresses cytotoxic T lymphocytes (CTLs) activity (9, 55). Mechanistically, lncEGFR specifically binds to EGFR and blocks its ubiquitination by c-CBL, leading to EGFR stabilization and to augment its activation and its downstream AP-1/NF-AT1 axis. Indoleamine 2, 3-dioxygenase (IDO) is encoded by *IDO1* gene and a heme-containing enzyme physiologically expressed in a number of tissues. IDO not only suppresses anti-tumor immune response in the body (56, 57), it also limits T-cell function and engages in mechanisms of immune tolerance (58). In breast cancer, small nucleolar RNA host gene 1 (SNHG1) negatively regulates the protein level of IDO to inhibit Treg cells differentiation and impede immune escape (29). In addition, lnc-SGK1 (Serine/threonine-protein kinase-SGK1) promotes Th2 and Th17 differentiation by SGK1/JunB signaling in gastric cancer (59). In HCC, Nuclear Enriched Abundant Transcript 1 (NEAT1) promotes CD8^+^ T cell apoptosis and enhances cytolysis through miR-155/Tim-3 pathway (30). Lnc-Tim-3 specifically binds to Tim-3 and blocks its interaction with Bat3, thus suppressing downstream Lck/NEAT1/AP-1 signaling and exacerbating CD8^+^ T cell exhaustion (32). NeST (Nettoie salmonella pas Theiler’s) is a lncRNA expressed in different immune cells including CD8^+^ T cells, CD4^+^ T T_H1_ cells, and natural killer cells (34, 60, 61). NeST promotes IFNG transcription to improve INF-γ production during CD8^+^ T cell activation (31). These findings indicate lncRNAs modulate immune cell priming and activation by different mechanisms. The emerging roles of lncRNAs in immune cell priming and activation indicate that these lncRNAs can be potential targets for cancer therapies.

## Role of lncRNAs in Immune Cell Migration

Recent studies have shown that some lncRNAs (e.g., lnc-sox5 and lincR-Ccr2-5’AS) are involved in immune cell trafficking. Knockdown of lnc-sox5 induces down-regulation of IDO1 in xenograft mouse model, leading to the modulation of CD3^+^CD8^+^ T cells infiltration and cytotoxicity (33). lincR-Ccr2-5’AS is a lncRNA specifically expressed in Th2 cells and transcribed in the opposite direction of the Ccr2 gene. Knockdown of lincR-Ccr2-5’AS induces a dramatic Th2 cells migration into the lung in mice (61). These findings spike the notion that lncRNAs can directly modulate the migration of immune cells.

## lncRNAs in Immune Cell Infiltration

Effective response of cancer immunity is also indispensable of effective T cells infiltrating into tumor stroma. By far, there are limited pieces of evidence about lncRNAs in the process of T cell infiltration in the cancer immunity cycle. In triple-negative breast cancer (TNBC) of patients receiving pembrolizumab (anti-PD-1) treatment, the pembrolizumab responders exhibited a relatively lower expression of LINK-A and a higher CD8^+^ T cell infiltration compared with non-responders (45), concluding that the CD8^+^ T cell infiltration in TNBC is negatively correlated with LINK-A expression (45). In breast cancer, lncRNA T-cell leukemia/lymphoma 6 (TCL6) is correlated with the infiltration of CD8^+^ T cells, CD4^+^ T cells, neutrophils, DCs, and the frequency of tumor-infiltrating lymphocytes (TILs) (35). Thus, more efforts to show direct evidence of whether and how lncRNAs function in immune cell infiltration are in urgent need.

## lncRNAs in the Recognition and Attack of Cancer Cells

Cell-cell recognition ability helps cells distinguish one type of neighboring cells from another. This ability requires complementary molecules of the different cellular surfaces to recognize each other, such as a receptor and its specific ligand. For example, programmed cell death 1 (PD-1) is an immune checkpoint receptor on the T cell surface. PD-1 binds to its ligands, programmed cell death 1 ligand 1 (PD-L1) and PD-L2 on cancer cell surface, functionally causing immune inhibition (17, 62). Overexpression of PD-L1 associates with poor prognosis and enables cancer cells to escape from immune surveillance in various cancer types. Of note, lncRNAs, including lncRNA AFAP1-AS1 and lncMX1-215, have been found to regulate PD-1/PD-L1 signaling. LncRNA AFAP1-AS1 can induce increased expression of PD1 in tumor microenvironment of nasopharyngeal carcinoma (36). Moreover, there is a positively expressing correlation between lncRNA AFAP1-AS1 and PD1 in nasopharyngeal carcinoma (NPC) (36). LncMX1-215, a novel IFNα-induced long noncoding RNA, significantly inhibits IFNα-induced expression of PD-L1 and galectin-9 in head and neck squamous cell carcinoma (37). In addition, in diffuse large B-cell lymphoma, MALAT1 sponges miR-195 to upregulate PD-L1 expression to promote cancer cells migration and immune escape. Inhibition of MALAT1 can rescue these events and attenuate epithelial-mesenchymal transition-like process (38). In esophageal squamous cell carcinoma, Small nucleolar RNA host gene 20 (SNHG20) promotes PD-L1 expression *via* ataxia telangiectasia mutated kinase (ATM)/JAK-PD-L1 pathway (39). In gastric cancer, UCA1 (Urothelial Cancer-Associated 1) promotes PD-L1 expression by repressing miR-26a/b, miR-193a, and miR-214, which contributes to immune escape of gastric cancer cells (40). These findings point out that lncRNAs are involved in the regulation of immune checkpoints in cancer immunity cycle, indicating that these lncRNAs may be synergistic targets for immunotherapy.

## Discussion

Immunotherapy has gained a lot of interest from researchers and clinicians due to its great potential for effectively treating cancer. The immune checkpoint inhibitors in the clinic, like PD1, PD-L1, and CTAL-4, have made great clinical achievements (63, 64). But some of the cancer patients do not respond to these immune checkpoint inhibitors, pointing out the need for more investigation of the cancer immune system. LncRNAs exhibit multiple and powerful functions in the tumor microenvironment, including tumorigenesis, tumor metastases, and immune disorders (10, 14, 33, 37). LncRNAs interact with DNA, mRNA, ncRNAs, and proteins to alter cellular physiology. LncRNAs have high tissue- and cell-specificity indicating a wholely different range of diagnosis and treatment of cancer (65). Some combination therapies, including combination of immunotherapy with chemotherapy, combination of surgery with chemotherapy, and even drug combination therapy, have attained obvious clinal progress (18, 62, 64, 66). Thus, consideration of a combination of targeting on lncRNAs and immunotherapy is attractive for cancer treatment. With the rapid development of RNA-targeting technologies, there are many approaches targeting lncRNAs emerging, like aptamers, small molecule inhibitors, natural antisense transcripts (NATs), an antisense ligonucleotide (ASO), synthetic lncRNA mimics, and other constructs (67). The off-target effect in systemic nucleic acid based therapies is a main problem in lncRNA therapies. CRISPR-Cas13a, a programmable RNA editing system that allows for temporal modulation of genetic variants in transcripts, may be a potential candidate for manipulation RNA expression with low off-target efficiency in lncRNA therapies. These approaches enable us to target lncRNAs to modulate their functions for clinical purposes. However, as for utilizing lncRNAs to aid in cancer diagnosis and therapy, the biggest challenge in lncRNAs research is the lack of conservation for many lncRNA species. Some cancer-related lncRNAs are well conserved, but most human cancer-related lncRNAs do not exhibit sequence conservation across mammalian species. This will impede preclinical research for therapeutic targeting in animals. By far, the patient-derived tumor organoids serve as a powerful model to explore the role of lncRNA in a patient-specific manner. Moreover, due to the complexity of the immune system, the functional investigation of lncRNAs in cancer immunity is just beginning, and their roles as both biomarkers and targets in cancer immunity cycle need further study.

## Author contributions 

AL contributed to the study design and data analysis, and edited the manuscript. MW and PF wrote the manuscript. MW and LQ contributed to the figure and table design. AL and JL edited the manuscript. All authors contributed to the article and approved the submitted version.

## Funding

This work was supported in part by the National Natural Science Foundation of China (81672791, 81872300), Zhejiang Provincial Natural Science Fund for Distinguished Young Scholars of China (LR18C060002) to AL. AL is a scholar of Thousand Youth Talents-China, a scholar of Thousand Talents-Zhejiang, a scholar of Hundred Talents-Zhejiang University.

## Conflict of Interest

The authors declare that the research was conducted in the absence of any commercial or financial relationships that could be construed as a potential conflict of interest.

## Abbreviations

LncRNA, long noncoding RNA; APC, antigen presenting cell; CTL, cytotoxic T lymphocyte; MHC I, major histocompatibility complex class I; NK, natural killer; NSCLC, non-small-cell lung cancer; PD1, programmed cell death protein 1; PD-L1, programmed cell death ligand 1; TCR, T cell receptor; TH, T Helper; TNBC, triple negative breast cancer; Treg, T-regulatory; CALR, calreticulin; VLDLR, very low-density lipoprotein-receptor; HCC, hepatocellular carcinoma; DC, dendritic cell; IDO, indoleamine 2, 3-dioxygenase; SGK1, serine/threonine-protein kinase; SNHG1, small nucleolar RNA host gene 1; NEAT1, nuclear Enriched Abundant Transcript 1; NeST, nettoie salmonella pas Theiler’s; TILs, tumor-infiltrating lymphocytes; NATs, natural antisense transcripts; ASO, antisense oligonucleotide.
